# Placental Hofbauer Cell Polarization Resists Inflammatory Cues In Vitro

**DOI:** 10.3390/ijms21030736

**Published:** 2020-01-22

**Authors:** Carolin Schliefsteiner, Sandra Ibesich, Christian Wadsack

**Affiliations:** Department of Obstetrics and Gynecology, Research Facility, Medical University of Graz, 8036 Graz, Austria; carolin.schliefsteiner@medunigraz.at (C.S.); sandra.ibesich@gmx.at (S.I.)

**Keywords:** Hofbauer cells, macrophages, placenta, inflammation, polarization, phenotype

## Abstract

Feto-placental Hofbauer cells (HBCs) are macrophages residing in placental stroma. They are generally described as anti-inflammatory M2 polarized cells, promoting tolerance and tissue remodeling. In certain pathologies, however, a possible phenotypical switch towards pro-inflammatory M1 macrophages has been proposed. The study aimed to determine if HBCs can acquire an M1 phenotype under pro-inflammatory conditions in vitro. HBCs were isolated from healthy human term placentas. Cells were cultivated upon addition of LPS and INF-γ or IL-4 and IL-13 to induce the M1 and M2 phenotype, respectively. Specific cell polarization markers and cytokines, associated with respective phenotypes, were investigated by flow cytometry and ELISA. THP-1 macrophages served as positive control. Pro-inflammatory stimuli reduced M2 markers CD163 and DC-SIGN, but did not induce M1 markers. TNF-α release was increased, but at the same time TGF-β and IL-10 release was upregulated, resembling in part the M2b sub-phenotype. Anti-inflammatory stimuli had no effect on HBC polarization. HBCs maintain their M2 phenotype in vitro despite inflammatory stimuli, which might represent a state of adaption and tolerance to avoid rejection of the semiallogeneic feto-placental unit.

## 1. Introduction

Macrophages belong to the key players of our immune system. They can eliminate pathogens by phagocytosis as first defense mechanism, but they also closely communicate with other immune cells to orchestrate an appropriate response to inflammatory events and tissue damage, either promoting inflammation or tissue homeostasis. Therefore, similar to the Th1 and Th2 phenotypes of T-cells, a phenotypical classification of macrophages has been proposed and macrophages are commonly distinguished into classically activated, pro-inflammatory M1 macrophages or alternatively activated anti-inflammatory M2 macrophages [1,2].

M1 macrophages are induced by agents such as LPS, INF-γ, and oxidized lipoproteins. They express M1-specific cell surface markers, e.g., CD80, CD86, CD40, and high levels of MHC class II proteins (MHC-II^high^). They secrete pro-inflammatory cytokines, such as IL-1, IL-12, and TNF-α. The primary function of M1 macrophages is host defense by phagocytosis.

M2 macrophages are induced by various compounds e.g., IL-4, IL-13, IL-10, and TGF-β but also glucocorticoids and TLR agonists. They express surface markers such as CD163, CD206 and DC-SIGN and only intermediate or low levels of MHC-II proteins (MHC-II^low^). Cytokines secreted by M2 macrophages include TGFβ, IL-10, and IL-1 among others. Their primary functions are tissue repair, wound healing and angiogenesis, as well as tolerance induction.

Of note, the classification into M1 and M2 macrophages is an over-simplification; current research indicates that macrophages can be activated within a broad spectrum between M1 and M2 macrophages [3], and M2 macrophages have been subdivided into further categories called M2a, M2b, M2c, and M2d [4]. With on-going research, other macrophage phenotypes such as pro-inflammatory M4 macrophages and atherosclerosis specific cell subsets are now emerging [5].

Human macrophages of the placenta, so-called Hofbauer cells (HBCs) are grossly vacuolated cells that can be of round, spindle-like, or stellate shape [6,7]. Their fetal origin has been demonstrated by sex chromatin staining [8]. In healthy pregnancies, HBCs are generally considered M2 polarized macrophages. This is supported by studies which demonstrated abundance of surface markers such as CD163, CD206, and DC-SIGN [9,10], and release of IL-10 and TGF-β [11]. Also DNA methylation profiling of HBCs has revealed tight regulation of M2 specific gene promoters [12].

HBCs are plastic cells and seem to fulfil various functions. These functions also correspond to the M2 phenotype rather than the M1 For instance, HBCs have been implicated in maternal tolerance towards the fetus [13] and in placental tissue remodeling, especially with respect to formation of blood vessels [14,15]. As immune cells, they are also implied in vertical transmission of pathogens from mother to child [16,17].

While it was supposed that inflammatory pathologies of the placenta, e.g., chorioamnionitis (CAM) or villitis of unknown etiology (VUE), might shift HBCs towards a more pro-inflammatory M1 phenotype, histopathological examination of placentas did not support these claims [10,18,19]. Regarding maternal metabolic disease, such as diabetes, there are conflicting findings: in patients with Type 1 diabetes mellitus (T1DM) HBCs might acquire an M1 phenotype [20], in patients with gestational diabetes mellitus (GDM), no switch from M2 to M1 seems to occur [21]. These two types of diabetes are, however, fundamentally different in their origin of disease and T1DM is characterized by potent inflammatory characteristics, whereas GDM only imposes a low-grade inflammatory environment. In Pre-eclampsia (PE), which is a pregnancy condition characterized by a higher degree of inflammation than diabetes, no data on HBC polarization but only on reduced HBC numbers exist [22]. In summary, it remains elusive if HBCs indeed adjust to inflammatory events during pregnancy or whether their phenotype is more stable than presumed, although it is known that macrophages usually adapt to their microenvironment.

This study aimed to determine if Hofbauer cells isolated from healthy human placentas at term can undergo a phenotypical switch from M2 to M1 polarization if stimulated accordingly in vitro. In comparison, a monocyte/macrophage cell line (THP-1) was used as positive control, proving that M1 and M2 phenotypes could be induced in vitro. The obtained data indicate that HBCs possess a steady and solid M2 phenotype which cannot be easily altered.

## 2. Results

### 2.1. Hofbauer Cells Do not Polarize Towards an M1 Phenotype upon LPS + INF-γ Treatment

**Figure 1:**
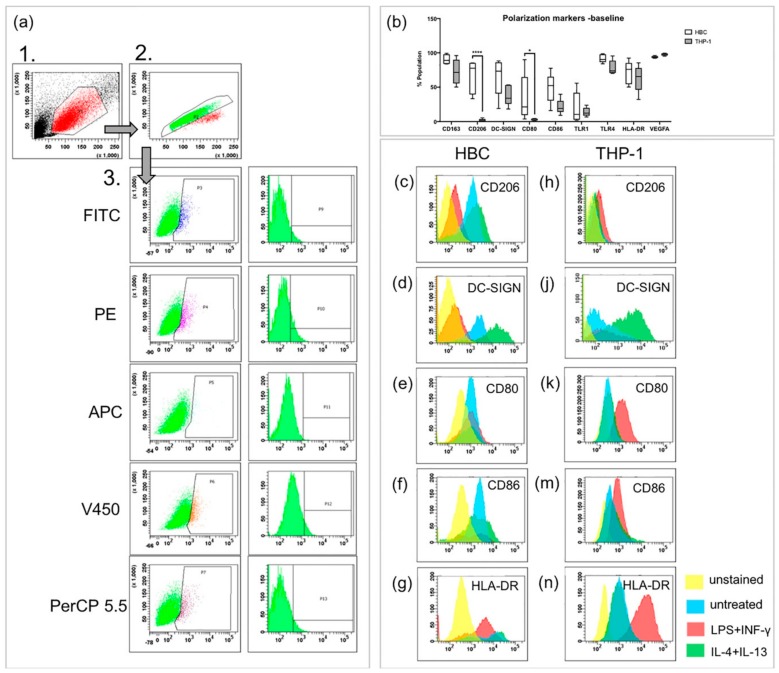
FACS analysis of polarization markers on Hofbauer cells (HBCs) and THP-1 cells. (**a**) Gating strategy in three steps, as performed in an unstained sample of HBCs. The same gating strategy was employed for THP-1 cells, minimally adjusting the third gate for the slightly different auto-fluorescence of THP-1 cells. (**b**) Comparison of marker expression on HBCs and THP-1 macrophages at baseline without treatment; *** *p*<0.001, * *p*=0.03. Box and whiskers plot represents data from *n* = 5 individual experiments per cell type depicted from minimum to maximum, the line within the box represents the median. (**c**,**d**) CD206 staining on HBC and THP-1 macrophages, respectively. (**e**,**f**) DC-SIGN staining on HBC and THP-1 macrophages, respectively. (**g**,**h**) CD80 staining on HBC and THP-1 macrophages, respectively. (**j**,**k**) CD86 staining on HBC and THP-1 macrophages, respectively. (**m**,**n**) HLA-DR (MHC-II) staining on HBC and THP-1 macrophages, respectively. Yellow histogram peaks represent unstained samples, blue represents unstimulated cells, red represents cells stimulated by LPS + INF-γ and green represents cells stimulated by IL-4 and IL-13. Histograms of one representative experiment are shown and for each cell type five individual experiments were conducted (*n* = 5).

Upon stimulation with LPS and INF-γ, macrophages usually become classically activated, presenting the M1 phenotype both in vivo and in vitro. FACS was used to study presence of extra-cellular and intracellular markers on HBCs and THP-1 cells. CD163, CD206, DC-SIGN, and VEGF were used as M2 markers, CD80, CD86, TLR1, and TLR4 were used as M1 markers. The MHC-II molecule HLA-DR was measured additionally since M1 macrophages have been described as MHC-II^high^ and M2 macrophages as MHC-II^low^ [23]. The three-step gating strategy employed for FACS experiments is shown in Figure 1a. First, cells were separated by size using forward and side scatter (FSC and SSC, respectively). Second, doublets were excluded by pulse-geometry gating the area and height of FSC. Lastly, the fluorescence signal of cells positive for respective markers was gated directly, plotting it against the SSC area by drawing polygonal gates.

Without any treatment, both HBCs and THP-1 macrophages contained cell populations that are positive for the above mentioned markers (Figure 1b). Interestingly, the M2 marker CD206 was almost absent on THP-1 macrophages, but present on about two thirds of HBCs (2% vs. 66% respectively, *p* < 0.001). Surprisingly, about one third of HBCs also expressed the M1 marker CD80 at baseline, but only 3% of THP-1 macrophages carried CD80 on their surface (*p* = 0.03). Generally, abundance of specific markers varied more on HBCs than on THP-1 macrophages; however, this can be explained by the fact that HBCs are primary cells prone to inter-individual differences, whereas THP-1 cells are an established immortalized cell line.

Interestingly, a decrease in cells positive for the two M2 markers CD206 (Figure 1c, *p* < 0.01) and DC-SIGN (Figure 1d, *p* < 0.05) was observed when HBCs were exposed to LPS and INF-γ. Nevertheless, HBCs apparently did not acquire a distinct M1 phenotype, as neither the numbers of cells positive for CD80 (Figure 1e), CD86 (Figure 1f), nor HLA-DR (Figure 1g) were significantly induced. In contrast, THP-1 macrophages exposed to LPS and INF-γ acquired an M1 phenotype which can be described as CD206^−^ (Figure 1h) and DC-SIGN^low^ (Figure 1j), but CD80^+^ (*p* < 0.05; Figure 1k), CD86^+^ (*p* < 0.05; Figure 1m) and HLA-DR^high^ (*p* < 0.001; Figure 1n). Conversely, upon stimulation of THP-1 macrophages with IL-4 and IL-13, which induces alternative activation, an increase in cells carrying DC-SIGN (*p* < 0.05, Figure 1j) was observed, and the population became HLA-DR^low^ (*p* < 0.05; Figure 1n). Both changes are indicative of M2 polarization of THP-1 cells. In IL-4 + IL-13 stimulated HBCs, no further changes compared to baseline were observed.

All obtained flow cytometry data are also summarized in Table 1 and graphical representation of the data is provided in Supplementary Figure S1.

### 2.2. LPS + INF-γ Stimulation Increases TNF-α and IL-12 but also IL-10 Release from HBCs

**Figure 2:**
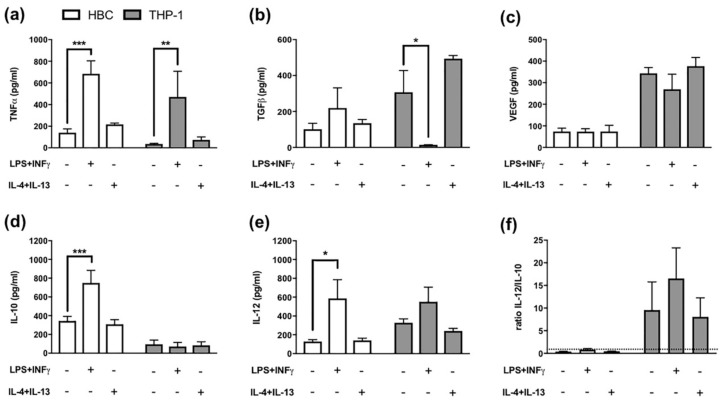
Cytokine Release from Hofbauer cells and THP-1 macrophages. (**a**) ELISA against TNF-α, *** *p* = 0.0005; ** *p* = 0.0045 (**b**) ELISA against TGF-β, * *p* = 0.02 (**c**) ELISA against VEGF, (**d**) ELISA against IL-10, *** *p* = 0.0007 (**e**) ELISA against IL-12, * *p* = 0.02 and (**f**) Ratio representing the proportion of IL-12 to IL-10 levels. The dotted line represents a ratio of 1. White bars = HBCs, grey bars = THP-1 macrophages. All data mean ± SD from 5 individual experiments each. Matched two-way ANOVA with Dunnett’s post-hoc test was used for statistical analysis.

The pattern of secreted cytokines released by macrophages additionally enables to differentiate between different M1 and M2 polarization stages. For instance, TNF-α has been described as pro-inflammatory cytokine released by M1 polarized macrophages, whereas TGF-β release is considered typical of M2 polarized macrophages [24]. When THP-1 macrophages and HBCs were treated with LPS and INF-γ, a significant increase in TNF-α release from both cell types was observed (*p* = 0.03 and *p* = 0.001, respectively; Figure 2a).

TGF-β release, on the other hand, was significantly reduced in THP-1 macrophages (*p* = 0.02; Figure 2b), but TGF-β release from HBCs was further increased, though not significantly (Figure 2b), when cells were exposed to pro-inflammatory conditions.

As only M2 macrophages have been demonstrated to possess pro-angiogenic properties [25], VEGF release from macrophages can serve as M2 marker. VEGF levels in both HBCs and THP-1 cells remained unaltered upon stimulation with either LPS plus INF-γ, or IL-4 plus IL-13. Of note, VEGF secretion of THP-1 cells exceeded that of HBCs more than three times independently of the stimulation (Figure 2c).

Furthermore, IL-10 and IL-12 release from HBCs and THP-1 cells after treatment was measured. The ratio of IL-12 to IL-10 can be used to distinguish between M1 and M2 polarized macrophages, the former being IL-12^high^/Il-10^low^ and the latter IL-12^low^/IL-10^high^. In HBCs, the release of IL-10 (*p* < 0.001; Figure 2d) and IL-12 (*p* = 0.02; Figure 2e) were both positively stimulated by LPS and INF-γ. In THP-1 macrophages, IL-10 release was generally low (Figure 2d), and only IL-12 release was stimulated by LPS + INF-γ exposure, however not significant (Figure 2e). After calculating the ratio of IL-12 to IL-10, HBCs remained IL-12^low^/IL-10^high^ even upon LPS + INF-γ treatment (Figure 2f), as the ratio was below 1. In THP-1 cells, the ratio yielded higher values (≥ 7) compared to HBCs (Figure 2f) and LPS + INF-γ treated THP-1 cells could be considered IL-12^high^/IL-10^low^ compared to untreated or M2-stimulated THP-1 macrophages (Figure 2f).

### 2.3. LPS + INF-γ Stimulation Alters Cell Morphology of HBCs but not THP-1 Cells

**Figure 3:**
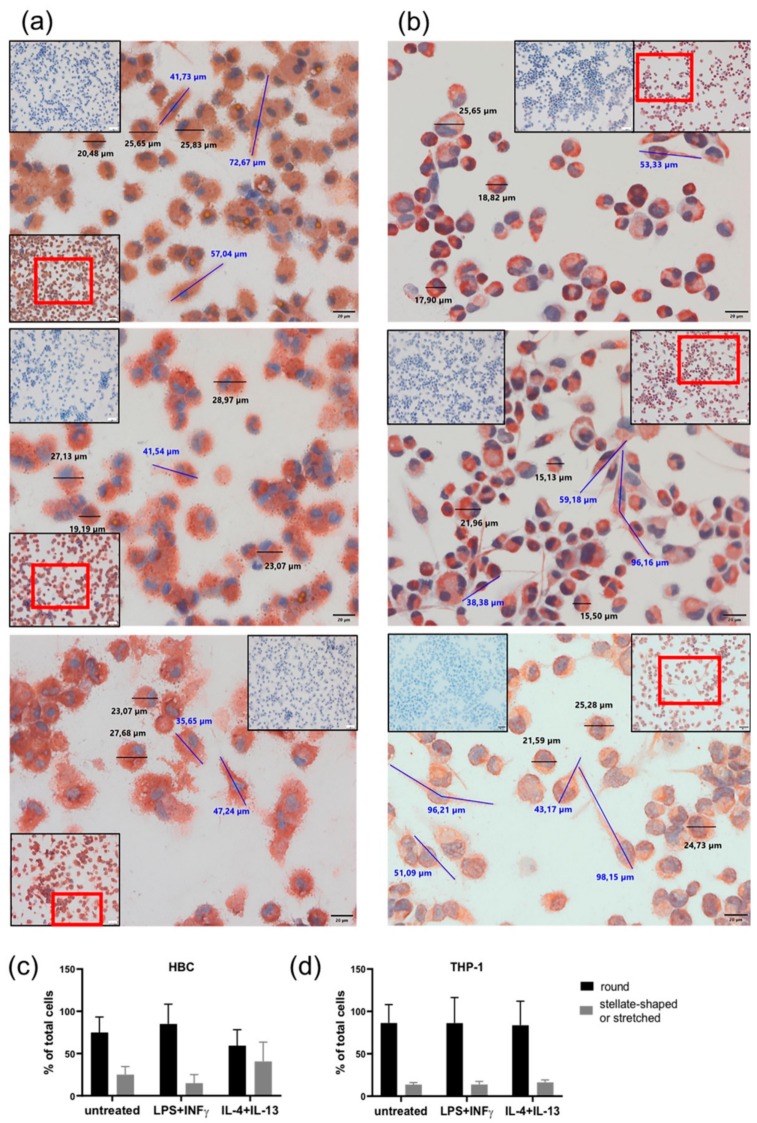
Changes in cell morphology upon phenotypical stimulation of macrophages. (**a**) HBCs exposed to no stimulus (top panel), LPS + INF-γ (center panel), or IL-4 + IL-13 (bottom panel). (**b**) THP-1 exposed to no stimulus (top panel), LPS + INF-γ (center panel), or IL-4 + IL-13 (bottom panel). Representative images out of 5 individual stainings per cell type are shown. Main pictures were taken at 40× magnification (scale bar = 20µm). Inserts show negative control stainings (blue) as well as a 20× magnification picture to provide a bigger picture, with the section chosen for 40× magnification in a red frame. Cell size was measured using CellSens Standard measuring tool. Representative round cells are marked by black bars and their respective size is given, elongated cells are marked by blue bars and their size is given. (**c**) Quantification of round and elongated or strellate-shaped HBCs upon stimulation compared to untreated control. (**d**) Quantification of round and elongated or stellate-shaped THP-1 upon stimulation compared to untreated control. For quantification, five different positions on a CD68 stained slide (*n* = 3 individual experiments/cell type) were photographed and cells counted.

Both HBCs and THP-1 macrophages were monitored closely by conventional light microscopy during all cell incubation experiments. Therefore, certain morphological changes upon treatment with LPS + INF-γ and IL-4 + IL-13 became apparent compared to unstimulated cells. Usually, right after isolation HBCs are round-shaped cells with prominent vacuoles in their cytosol. Within 48 to 72 h post-isolation, they stretch and become more spindle-like or even triangular, and eventually form tiny cell protrusions. Interestingly, we observed that over the course of the 72 h experimental period, HBCs at baseline and even more so IL-4 + IL-13 stimulated HBCs did acquire this morphological phenotype, whereas LPS + INF-γ stimulated HBCs remained small and round in shape. In contrast, THP-1 macrophages differentiated more uniformly; unstimulated THP-1 cells were more round in shape than stimulated cells, and LPS + INF-γ stimulated cells appeared similarly stretched out as those treated with IL-4 + IL-13. As part of the routine quality control procedure to check the purity of HBC isolations, immune cytochemistry is employed and cells were also stained (among other markers) against CD68, a marker for tissue macrophages independent of their polarization [26]. In support of our previous observations, CD68 stained HBCs appeared more stretched out with visible pseudopodia under control conditions (Figure 3a, top panel) or when stimulated with IL-4 + IL-13 (Figure 3a, bottom panel), whereas LPS + INF-γ stimulated HBCs appear round and granulose, lacking any pseudopodia (Figure 3a, center panel). THP-1 macrophages stained against CD68 again seemed more uniform, with some round cells and some stretched out cells presenting with pseudopodia independent of stimulation with respective agents (see Figure 3b, all panels). Little is known about the relationship of cell morphology and polarization state, but some studies claimed that a round shape is associated with an M2 phenotype, while spindle like shape is indicative of the M1 phenotype [27,28,29,30]. We took advantage of the possibility to measure cell size (diameter and length) using the CellSens software package. Independent of treatment, both HBCs and THP-1 cells ranged in size from about 22 ± 3 µm for round cells and 55 ± 15 µm for elongated cells (mean ± SD). Whereas, stretched cells were easily found in THP-1 cells treated with LPS + INF-γ, HBCs exposed to the same stimulus remained mostly round in shape. The total number of cells was assessed and the proportion of round and elongated cells was calculated relative to total cells. Although the changes were not significant, the obtained data show that our initial observation was true: in HBCs, the proportion of round cells is higher in the LPS + INF-γ treated group and the proportion of elongated ells is higher in IL-4 + IL-13 treated cells as compared to control (Figure 3c), whereas proportion of round and elongated cells is less variable in THP-1 macrophages upon treatment (Figure 3d).

## 3. Discussion

In this study, we investigated the potential of HBCs, tissue macrophages of the human placenta, to re-polarize in vitro and undergo a phenotypical switch. Although it has been demonstrated that macrophages generally are versatile cells, adapting readily to their environment in vivo, we only observed few changes to macrophage polarization upon stimulation of cells in vitro. Moreover, the observed changes upon pro-inflammatory stimulation do not clearly indicate re-polarization towards the M1 phenotype.

It is commonly accepted that HBCs stem from mesenchymal cells in the first trimester of pregnancy [6], but after the fetal circulation has been established HBCs differentiate from fetal monocytes recruited into placental tissue [31].

There are several hypotheses on how monocytes differentiate and polarize into M1 or M2 subsets. It seems most likely that depending on micro-environmental cues monocytes acquire either M1 or M2 properties. Porcheray et al. demonstrated that peripheral blood monocytes (PBMCs) differentiated to mature macrophages in vitro adapt to M1 stimuli and become M1 polarized, but can be re-polarized to M0 (naive) or even M2 macrophages, if switched to appropriate culture conditions. Furthermore, vice versa a switch from M2 to M0 or M1 is possible in vitro [27,32]. Furthermore, in tumor-associated macrophages (TAMs), which are default M2 polarized, repolarization from M2 to M1 macrophages has been observed when TAMs were stimulated with LPS or INF-ɣ [33,34]. Therefore, one might assume that also HBCs could be re-polarized in vitro under appropriate conditions.

We observed that HBCs at baseline, do not present only with M1 or only M2 surface markers. Within the HBC population, the majority of cells expressed the M2 markers CD163, CD206, and CD209, but also carried M1 markers CD80 and CD86. This data demonstrates the complexity of ascribing a defined phenotype to HBCs just at baseline. Amara et al. have investigated placental macrophages (decidual and HBCs), and studying marker panels, cytokine release, and phagocytic activity, concluded that placental macrophages might not be polarized at all [19]. This is an intriguing statement; nevertheless, many studies indicate presence of a Th2/M2-favoring environment in pregnancy (reviewed in [35,36]). Of note, THP-1 macrophages, used as comparative cell model for treatments, were not fully M1 or M2 at baseline, but also expressed CD163, CD209, and CD86 simultaneously.

A study investigating Toll-like receptor (TLR) signaling in HBCs found that these cells express high levels of TLR3 and TLR4 (the latter we demonstrated as well) and present with typical M2 surface markers, yet secrete high levels of pro-inflammatory IL-6 and IL-8 upon LPS treatment. It concluded that although HBCs are M2 polarized, they might exert pro-inflammatory features upon TLR activation [37]. Moreover, M2b macrophages are known to share properties of M1 macrophages, e.g., increased expression of CD86, as well as increased release of TNF-α but also high release of IL-10 [38]. In line with these accounts, we suppose that also LPS + INF-γ stimulated HBCs mostly maintain M2 features (e.g., low positivity for CD80, CD86, and MHC-II proteins; high release of IL-10), yet at the same time acquire certain features resembling the M1 or even more so the M2b phenotype (e.g., decrease in DC-SIGN positive cells; increased release of TNF- α and IL-12).

While it is hard to determine HBCs polarization after M1-induction, THP-1 macrophages upon LPS + INF-γ stimulation clearly acquired an M1 phenotype presenting with high numbers of cells positive for CD80 and CD86 as well as HLA-DR, secreting high levels of TNF-α, but low levels of TGF-β and IL-10. Induction of the M2-phenotype upon IL-4 + IL-13 treatment was represented by increased positivity for DC-SIGN and decreased staining against HLA-DR. Although recent studies claimed that e.g., higher concentrations of PMA used to differentiate THP-1 monocytes into macrophages might affect experimental outcome with respect to macrophage polarization [39,40], it seems that in our study THP-1 macrophages provided a proper cell model for testing treatment efficacy.

While it is an asset of this study that exclusively human sample material was used, there are some apparent limitations to this study. First, the selection of appropriate surface markers and cytokines representative of either the M1 or M2 phenotype is always a compromise. We are aware that other researchers might have included other markers in their panels. We opted to use CD163, CD206, and DC-SIGN as surface markers of the M2 phenotype, as well as TGF-β, IL-10, and VEGF release. These markers are well studied with respect to M2 macrophages but especially the reproductive organs and placenta [41,42]. As M1 markers, we chose the co-receptors for macrophage-induced T-cell activation, CD80 (B7-1) and CD86 (B7-2), as well as TNF-α and IL-12 as signature cytokines, which are all well-established M1 markers [3].

Second, our study focused on the presence of polarization markers and cytokine release. Although these parameters are commonly used when characterizing macrophage phenotype, they are mostly descriptive. We are aware that macrophages exhibit relevant physiological effector functions and that functional assays should be within the scope of our future studies. In previous studies, we did, however, investigate HBCs for their pro-angiogenic potential and found that conditioned media from these cells can induce tube and network formation in placental endothelial cells [43]. Of note, the ability to promote angiogenesis has been described in M2 polarized macrophages only [25].

Third, more and more researchers point out the difficulties investigating not only, but given their versatility, especially macrophages taken out of their in vivo situation and put into cell culture [44,45]. While one can cultivate HBCs after isolation from tissue, one can never substitute for all the compounds and factors making up their complex in vivo environment.

Therefore, our data has to be related to histological studies in whole placental tissue, especially in studies investigating inflammatory pregnancy pathologies. Unfortunately, many histological studies focused on markers such as CD14 and CD68, some included CD163, as it is well described for HBCs. CD14 is a co-receptor of TLR4, recognizing LPS and might therefore be considered an M1 marker, but it is also present on all monocytes and monocyte-derived macrophages, so it is not an ideal polarization marker. CD68 is also a pan-macrophage marker [26], and not related to either activation state. Thus, many studies focused on HBC numbers rather than activation.

Nevertheless, there are some useful studies to discuss with respect to HBC polarization. Joerink et al. investigated macrophage polarization in chorioamnionitis (CAM), caused by bacterial infection. They found that neither control nor CAM placenta stained positive for any of three selected M1 markers, but positive for all selected M2 markers (CD163, CD206, and DC-SIGN). Also on mRNA level the same observation was made. They concluded that CAM does not induce a switch towards M1 polarized HBCs [10]. In pre-eclamptic pregnancies, one study investigated CD163, CD68, folate receptor β (FR-β), CD206, and DC-SIGN in histology and on mRNA level. A dramatic decrease in all markers was observed [22]. However, no M1 markers were investigated, so one can only speculate if there is shift from M2 to M1 polarized macrophages or an absolute decrease in HBC numbers in pre-eclampsia. Another study investigated CD14 and CD68 in addition to CD163 and DC-SIGN with a similar outcome, a reduction in all markers was observed, pointing towards decreased HBC numbers in PE. However, this study also observed decreased IL-10 production, which might be related to decreased HBC numbers, but might also suggest a shift to a more pro-inflammatory HBC phenotype [46].

Furthermore, in a previous study on HBCs from our group, histological data on total placental tissue compared to FACS data of isolated HBCs complemented one another well, both demonstrating that HBCs numbers and M2 phenotype were unchanged by maternal gestational diabetes [21]. Also, Young and colleagues, using the same isolation protocol for HBCs as our studies, observed a close correlation of their histological findings and data obtained by FACS [37]. Therefore, it seems feasible to assume that HBCs isolated from tissue cultivated in vitro still possess most of the properties they also present in situ and that the findings obtained in the current study—showing that HBCs have a very stable phenotype which cannot be readily altered—relate to the actual situation in tissue in vivo.

However, why would HBCs maintain their M2 characteristics also under pro-inflammatory conditions?

Successful pregnancy requires accurate, timely regulation of immune-regulatory processes, macrophages are the predominant leukocyte population of in maternal uterine tissue [47] and fetal HBCs are estimated to account up to 40 percent of the placental villous mass [48,49]. Therefore, macrophages are of utmost importance at the feto-maternal interface. Throughout different stages in pregnancy predominance of either M1 or M2 macrophages has been observed and the balance between those subsets is crucial for successful pregnancy outcome. This was shown specifically in macrophages of the decidua and amnion [50,51] but also in HBCs [43]. Importantly, alterations in HBC activity have been associated with second trimester pregnancy loss [52] and glucocorticoids, which are used as medication against pre-term labor, alter HBC function via induction of CD163 [53]. Also, labor on-set is characterized by presence of M1 macrophages in decidua [54], while M2 polarized HBC numbers seem to decrease towards term as compared to first trimester [55].

Considering evolutionary principles and the importance of creating viable off-spring, it makes sense that macrophages of fetal origin interact with the maternal immune system in a way that propagates fetal survival. Therefore, a stable, homeostatic, and tolerance-inducing M2 phenotype that only adapts minimally to pro-inflammatory conditions might actually be desired in pregnancy. To answer this question and to further elucidate possible contributions of HBCs to pregnancy pathologies but also healthy placenta development, further studies of this pleiotropic cell type are needed.

## 4. Materials and Methods

### 4.1. Isolation of Hofbauer Cells

Placentas from healthy donors with uncomplicated singleton pregnancies were obtained within 20 min after delivery. Patient characteristics are shown in Supplementary Table S1. The study was approved by the institutional ethics committee of the Medical University of Graz (27-265 ex 14/15) and all mothers gave written informed consent. For HBC isolation, the decidua was removed to omit contamination with decidual macrophages. Villous tissue was cut, finely minced and stored overnight in PBS buffer. About 60 to 100 g tissue was used for isolation. Tissue was digested employing trypsin (10×, Gibco, Carlsbad, CA, USA), Collagenase A (Roche, Basel, Switzerland) and DNase I (Roche, Basel, Switzerland). Subsequently, cells were applied onto a Percoll gradient (Gibco, Carlsbad, CA, USA) and centrifuged at 1000 g for 30 min, without brake. HBCs emerged as band between the 30%–35% Percoll layers. Cells were collected from the gradient and negative immune selection using magnetic beads (Dynabeads anti-goat IgG, Invitrogen, Carlsbad, CA, USA) and antibodies against CD10 (abcam, Cambridge, United Kingdom) and EGFR (Millipore, Billerica, MA, USA) was used to purify the cells. HBCs were counted and plated in macrophage medium (MaM, ScienCell, Carlsbad, CA, USA) supplemented with 5% FCS and macrophage growth supplements (ScienCell, Carlsbad, CA USA) and antibiotics (Pen/Strep, Gibco, Carlsbad, CA, USA) at a density of 1 × 10^6^ cells/mL. HBCs were cultivated at 21% oxygen, 37 °C and allowed to settle in vitro for 12 h before treatment.

### 4.2. Cultivation of THP-1 Cells and Macrophage Differentiation

THP-1, a monocytic cell line derived from an acute monocytic leukemia patient, was obtained from ATCC and cultivated in RPMI-1640, supplemented with 10% FCS and antibiotics (all Gibco, Carlsbad, CA, USA) at 37 °C and 21% oxygen. THP-1 monocytes, growing in suspension, were differentiated into adherent macrophages by exposure to phorbol-12-myristate-13-acetate (PMA, Sigma, St. Louis, MO, USA) at a final concentration of 100 ng/mL for 4 h. Thereafter, THP-1 macrophages were washed once with HBSS (Gibco, Carlsbad, CA, USA) and cultivated in RPMI-1640 as described before. THP-1 macrophages were used subsequently for treatment.

### 4.3. Phenotypical Stimulation of THP-1 Macrophages and Hofbauer Cells

Treatment of HBCs was performed in complete MaM medium, treatment of THP-1 macrophages in complete RPMI-1640 and performed in T25 nunclone flasks (Nunc, Waltham, MA, USA) at a cell density of 1 × 10^6^ cells/mL. For induction of polarization towards M1 phenotype, lipopolysaccharide (LPS, Sigma, St. Louis, MO, USA) and Interferon gamma (INF-γ, Sigma, St. Louis, MO, USA) were added to a final concentration of 50 and 20 ng/mL, respectively. For induction of the M2 phenotype, interleukin 4 and 13 (IL-4, IL-13, both Sigma, St. Louis, MO, USA) were added to a final concentration of 20 ng/mL each. Both cell types were cultivated for 72 h after addition of stimulating agents. Untreated controls were included in each individual experiment. THP-1 macrophages were harvested after 72 h treatment and used for subsequent analysis. HBCs received a second treatment with stimulating substances and were cultivated for an additional 24 h before harvesting and proceeding. Preliminary experiments on HBCs from our group and also others [56] have shown that the isolation protocol using extensive digestion steps can reduce presence of surface markers in FACS and that up to 72 h recovery period are needed before e.g., CD163 is fully present again. Therefore it was decided to offer HBCs more time in culture exposed to respective stimuli.

### 4.4. Flow Cytometry

Macrophages were carefully harvested using accutase enzyme solution and gentle scraping. Cells were counted and a minimum of 3 × 10^5^ cells was used for each FACS staining; viability after detachment by accutase and scraping usually ranged between 65% and 75% and both cell number and viability were determined using a CASY cell counter model TT (Innovatis, Bielefeld, Germany). Fc-receptors were blocked in 3% BSA (Sigma, St. Louis, MO, USA) in 1 × HBSS for 10 min. If needed, for intra-cellular targets, cells were permeabilized and fixated using Perm-/Fix-Solution (BD). Otherwise, cells were re-suspended in PBS and incubated with the respective antibodies for 30 min at 4 °C. Antibodies used for FACS are provided in Supplementary Table S2. Unstained and total IgG stained cells were used as controls. Cells were washed twice after antibody incubation and re-suspended in PBS for counting. Cell sorting was performed on an LSR-II instrument (BD Bioscience, Franklin Lakes, NJ, USA), using FACSDiva software v.8 both for acquisition and analysis. Cells were gated in three steps; first, cells were separated by size using forward and side scatter (FSC and SSC, respectively). Second, doublets were excluded by pulse-geometry gating the area and height of FSC. Lastly, the fluorescence signal of cells positive for respective markers was gated directly, plotting it against the SSC area by drawing polygonal gates. FACS data are represented as % positive population; although reporting data using MFI is also a common approach, we consider this the appropriate parameter, as the same cell type, or in fact the same primary cell isolation for HBCs, was stimulated and compared for marker expression to unstimulated cells.

### 4.5. Enzyme Linked Immunosorbent Assays

Cell culture supernatants from HBCs and THP-1 macrophages were collected when cells were harvested, i.e., 72 h post stimulation, and used to detect secreted cytokines representative for either M1 or M2 phenotype. In consideration of the limit of detection (LOD) of respective ELISAs, samples were four-fold concentrated, using Vivaspin centrifugal filter columns with a molecular weight cut-off of 3 kDa (Sartorius, Göttingen, Germany). Levels of TNF-α, IL-10, IL-12, and VEGF (all Peprotech, Rocky Hill, NJ, USA) as well as TGF-β (eBioscience, San Diego, CA, USA) were quantified following the manufacturer’s instructions. Data were obtained using a SpectroStar Nano plate reader (BMG Labtech, Ortenberg, Germany) and quantification performed using Mars Data Analysis Software v3.32 (BMG Labtech, Ortenberg, Germany).

### 4.6. Immune Cytochemistry (ICC)

HBCs and THP-1 cells were plated and treated as described before on 4-well glass chamber slides (Nunc Lab-Tek, Waltham, MA, USA), in order to carry out ICC using isotype controls and reagents from Dako. Cells were washed twice with HBSS and fixed with ice cold acetone. Antibodies were diluted to working concentration using Dako antibody diluent and cells were incubated with primary antibodies for 30 min. Cells were subsequently incubated with Dako antibody enhancer for 10 min. After a washing step, cells were incubated with large HRP polymer solution and AEC chromogen solution, for 20 and 10 min, respectively. Negative cells and nuclei were counterstained with hematoxylin solution (Sigma, St. Louis, MO, USA) and mounted with glycerin (Sigma, St. Louis, MO, USA). Antibodies against the pan-macrophage marker αCD68 (Dako, Santa Clara, CA, USA, mouse monoclonal clone KP1, diluted 1:100) and goat-α-mouse-IgG (Dako, Santa Clara, CA, USA, dil. 1:200) as isotype control were used in ICC stainings. Images were obtained using a bright filed microscope equipped with an Olympus camera operated by CellSens Standard software. For statistical purposes, CD68 staining on HBCs and THP-1 cells was evaluated in three independent experiments for each cell type. Photographs of four different areas on the slide were taken. The number of round and elongated cells was counted and expressed in proportion to the total cell number. Cell diameter/size of representative cells was measured using the measuring tool provided within the CellSens Standard software package and cellular size of round and elongated cells was expressed as mean ± SD.

### 4.7. Statistical Analysis

GraphPad Prism v8 (Graphpad Software, San Diego, CA, USA) was used for all statistical calculations and plotting of graphs. Shapiro–Wilk-test was used to test for normal distribution. Two-way ANOVA with Dunnett’s post-hoc test to correct for multiple comparisons was used to calculate differences between treatment groups. *p*-values below 0.05 were considered as statistically significant.

## 5. Conclusions

In conclusion, our data show that HBCs at baseline are neither clearly M1 nor M2 polarized, however, they still appear more M2-like than M1-like even under pro-inflammatory conditions. As the M1/M2 paradigm is currently being challenged and new distinct phenotypes along a possible spectrum of macrophage polarization emerge (e.g., M_ox_, M_heme_, and M4), it may be time to revisit HBC polarization and maybe introduce a placental macrophage phenotype (which could e.g., be termed M_plac_). However, as our study is of piloting character given its small sample size, HBC polarization has to be determined in larger patient groups in the future to substantiate our findings.

## Figures and Tables

**Table 1:** Percentage of HBC and THP-1 macrophages positive for indicated polarization markers upon M1 and M2 stimulation in comparison to untreated cells. For each cell type, five individual experiments were conducted. In the case of HBCs the five individual experiments equal five different cell isolations, i.e., donors. 2-way ANOVA with Dunnett’s post-hoc test was used to test statistical significance.

	HBC% pos. Population				THP-1% pos. Population			
	Untreated	LPS + INF-γ	IL-4 + IL-13	*p*-Value	Untreated	LPS + INF-γ	IL-4 + IL-13	*p*-Value
CD163	82.9 ± 13.7	67.7 ± 31.5	79.0 ± 11.5		70.3 ± 24.7	91.5 ± 4.9	78.0 ± 18.4	
CD206	76.7 ± 18.4	27.6 ± 6.6**	81.0 ± 4.7	** 0.01	2.7 ± 1.5	6.2 ± 0.7*	2.7 ± 1.7	*0.02
DC-SIGN	77.7 ± 11.4	26.9 ± 16.4*	93.1 ± 2.7	* 0.03	41.4 ± 10.5	34.7 ± 15.6	57.0 ± 32.2*	*0.01
CD80	40.4 ± 20.4	32.8 ± 14.0	42.0 ± 20.2		4.9 ± 3.5	46.9 ± 18.5*	3.2 ± 1.9	*0.03
CD86	67.6 ± 18.1	50.1 ± 14.0	72.8 ± 11.3		24.8 ± 8.1	61.2 ± 19.7*	23.1 ± 9.8	*0.02
TLR1	31.4 ± 24.4	26.6 ± 11.3	34.7 ± 19.3		14.1 ± 5.7	16.1 ± 5.3	7.7 ± 3.6*	*0.04
TLR4	96.0 ± 3.6	84.6 ± 13.1	91.7 ± 4.3		74.3 ± 14.1	93.2 ± 12.0	66.3 ± 16.5	
HLA-DR	74.8 ± 16.1	62.8 ± 12.8	77.5 ± 10.4		64.9 ± 13.7	95.8 ± 7.2**	43.8 ± 8.9*	**0.005; *0.025
VEGF	94.4 ± 6.5	88.8 ± 7.9	92.1 ± 7.8		96.8 ± 1.8	99.3 ± 0.5	96.8 ± 2.5	

## Supplementary Materials

Supplementary Materials can be found at https://www.mdpi.com/1422-0067/21/3/736/s1.

## Abbreviations

HBCs	Hofbauer cells
LPS	Lipopolysaccharid
TNF-α	Tumor necrosis factor alpha
TGF-β	Transforming growth factor beta
INF-ɣ	Interferon gamma
IL-	Interleukin
TAM	Tumor associated macrophage
CAM	Chorioamnionitis
VUE	Villitis of Unknown Etiology
T1DM	Type 1 Diabetes Mellitus
GDM	Gestational Diabetes Mellitus
