# Supplementary material for: Placental Hofbauer Cell Polarization Resists Inflammatory Cues In Vitro
Source: Int J Mol Sci. 2020 Jan 22;21(3):736. doi: 10.3390/ijms21030736 (PMC7038058; doi:10.3390/ijms21030736)
Supplement: Supplementary file 1 [file ijms-21-00736-s001.pdf]

International Journal of
Molecular Sciences

MDPI

Supplemental Material for:

Placental Hofbauer Cell Polarization Represents A Stable Phenotype Which Cannot Be Altered Readily *In Vitro*

Carolin Schliefsteiner[1,†], Sandra Ibesich[1,†] ,Christian Wadsack[1,*]

[1] Department of Obstetrics and Gynecology, Research Facility, Medical University of Graz – sandra.ibesich@sgmx.at; carolin.schliefsteiner@medunigraz.at

* Correspondence: christian.wadsack@medunigraz.at

† These authors contributed equally to the present work

Received: date; Accepted: date; Published: date

Supplementary Material

Patient Characteristics	
Maternal Age¶ (years)	31 (28-34)
Maternal BMI before pregnancy (kg/m^2)	21.2±1.4
Maternal BMI at delivery (kg/m^2)	26.6±1.7
Placental Weight (g)	578.0±61.8
Neonatal weight (g)	3276.8±456.9
Neonatal lenght (cm)	51.0±2.6
Neonatal ponderal index (kg/m^3)	2.5±0.2
Mode of delivery*	CS=2, V=3
Gestational Age¶ (wks)	39 (37-40)

*CS= cesearean section, V = spontaneous vaginal delivery

¶ all data mean±SD except Maternal and Gestational Age which is presented as median (range)

Supplementary Table 1 Obstetrical and biometric characteristics of study subjects whose placenta was used for HBC isolation (n=5).

Antibody against	Company	Catalogue Number	Clone	Fluorophor	Dilution
CD163	BioLegend	#333610	GHI/61	APC	1:7
CD206	BD	# 551135	19.2	FITC	1:10
DC-SIGN	BD	# 558263	DCN46	PerCP5.5	1:10
CD80	BD	# 560444	L307.4	V450	1:10
CD86	BD	#560359	2331	V450	1:10
TLR-1	Abcam	#ab59702	GD2.F4	FITC	1:20
TLR-4	RnDSystems	#FAB6248P	610015	PE	1:20
HLA-DR	BioLegend	#307635	L243	V450	1:20
VEGF	RnDSystems	#IC2931P	23410	PE	1:20

Supplementary Table 2 Antibodies used in FACS experiments. All antibodies used were monoclonal antibodies generated in mice.

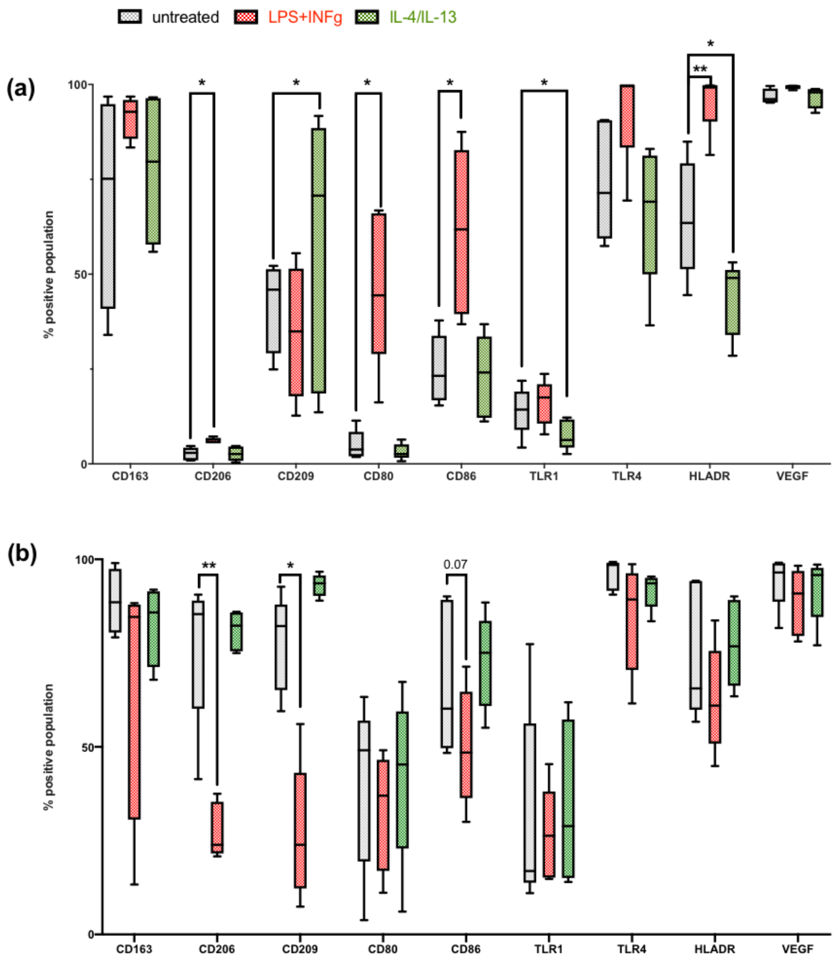

Supplementary Figure 1 (a) Changes in polarization marker expression in THP-1 macrophages upon stimulation with LPS+INF-γ (red) and IL-4/IL-13 (green). Unstimulated cells are shown in gray. (b) Changes in polarization marker expression in HBCs upon stimulation with LPS+INF-γ (red) and IL-4/IL-13 (green). Unstimulated cells are shown in gray. Box and whiskers plot represents the data from minimum to maximum values (whiskers) and the line within the box represents the median. Data from five individual experiments per cell type were pooled (n=5) and statistical significance was tested using 2-way ANOVA with Dunnett's post-hoc test correcting for multiple comparisons.

© 2020 by the authors. Submitted for possible open access publication under the terms and conditions of the Creative Commons Attribution (CC BY) license (http://creativecommons.org/licenses/by/4.0/).
